# Mechanism of Mercury Adsorption and Oxidation by Oxygen over the CeO_2_ (111) Surface: A DFT Study

**DOI:** 10.3390/ma11040485

**Published:** 2018-03-23

**Authors:** Li Zhao, Yangwen Wu, Jian Han, Qiang Lu, Yongping Yang, Laibao Zhang

**Affiliations:** 1 National Engineering Laboratory for Biomass Power Generation Equipment, North China Electric Power University, Beijing 102206, China; Zhaoli9533@163.com (L.Z.); zorowuyangwen@163.com (Y.W.); hj19940929@163.com (J.H.); yyp@ncepu.edu.cn (Y.Y.); 2 Cain Department of Chemical Engineering, Louisiana State University, Baton Rouge, LA 70820, USA; laibaozhang21@gmail.com

**Keywords:** Hg^0^ oxidation mechanism, surface oxygen, CeO_2_ (111) surface, DFT study

## Abstract

CeO_2_ is a promising catalytic oxidation material for flue gas mercury removal. Density functional theory (DFT) calculations and periodic slab models are employed to investigate mercury adsorption and oxidation by oxygen over the CeO_2_ (111) surface. DFT calculations indicate that Hg^0^ is physically adsorbed on the CeO_2_ (111) surface and the Hg atom interacts strongly with the surface Ce atom according to the partial density of states (PDOS) analysis, whereas, HgO is adsorbed on the CeO_2_ (111) surface in a chemisorption manner, with its adsorption energy in the range of 69.9–198.37 kJ/mol. Depending on the adsorption methods of Hg^0^ and HgO, three reaction pathways (pathways I, II, and III) of Hg^0^ oxidation by oxygen are proposed. Pathway I is the most likely oxidation route on the CeO_2_ (111) surface due to it having the lowest energy barrier of 20.7 kJ/mol. The formation of the HgO molecule is the rate-determining step, which is also the only energy barrier of the entire process. Compared with energy barriers of Hg^0^ oxidation on the other catalytic materials, CeO_2_ is more efficient at mercury removal in flue gas owing to its low energy barrier.

## 1. Introduction

Mercury, a pollutant harmful to human health and the environment, has become a global concern due to its toxicity, high volatility, and bioaccumulation [1,2,3,4]. In October 2013, the first legally-binding international treaty, called the Minamata Convention, was concluded to limit global emissions of mercury [5,6]. The convention entered into force on 16 August 2017. Coal-fired power plants are regarded as major sources of atmospheric mercury emissions [7,8]. The latest version of the Emission Standard of Air Pollutants for Thermal Power Plant (GB13223-2011), released by the Ministry of Environmental Protection of China, set the limit for the emission of mercury and its compounds from coal-fired boilers to 0.03 mg/m^3^. Therefore, mercury removal technology for coal-fired flue gas is urgently needed.

Mercury in coal-fired flue gas mainly exists in three forms, i.e., elemental mercury (Hg^0^), oxidized mercury (Hg^2+^), and particle mercury (Hg^P^) [9]. Hg^2+^ is soluble in water and can be removed primarily in the form of HgCl_2_ by the wet desulfurization system (WFGD) in coal-fired power plants [10]. Hg^P^ can be removed with the fly ash in fabric filters (FF) and electrostatic precipitators (ESP) [11]. Hg^0^ is the main form of mercury released into the atmosphere, and is difficult to remove using existing pollutant control equipment in power plants due to its chemical inertness and insolubility in water [12]. The main solution for Hg^0^ removal is by the adsorption and oxidation method [13]. The injection of activated carbon or other adsorbents for Hg^0^ adsorption in flue gas greatly increases the cost of power plant because of the limited capacity and high cost of the adsorbent. On the other hand, mercury’s oxidation to readily captured Hg^2+^ by catalyst needs no installation of new equipment and can save significant transformation and operating costs compared to sorbents injection, which makes it easier to accept [14,15]. Hence, the development of efficient Hg^0^ oxidation catalysts is of great significance. 

CeO_2_ is a promising catalytic oxidation material with low cost, no toxicity, and a large oxygen storage capacity because it contains the unique redox couple Ce^3+^/Ce^4+^, which can shift from CeO_2_ to Ce_2_O_3_ and vice versa, under oxidizing and reducing conditions, respectively [16,17]. Experimental results from Li et al. and Fan et al. showed that CeO_2_ had a satisfactory catalytic performance in the oxidation of Hg^0^ and maintains over 90% oxidation efficiency under simulated flue gas conditions [16,18]. Zhao et al. investigated the modification of a commercial SCR catalyst with a series of metal oxides and found that the SCR catalyst doped with CeO_2_ exhibited the highest Hg^0^ oxidation ability [19]. Theoretically, Liu et al. employed the DFT method to study the reaction mechanism of Hg^0^ oxidation by HCl on the CeO_2_ (111) surface, verifying the catalytic activity of CeO_2_ for Hg^0^ oxidation theoretically by analyzing energy barriers. It was found that the energy barrier of forming HgCl_2_ on the CeO_2_ surface was close to that on noble metals such as Au and Pd. The low energy barrier makes CeO_2_ an attractive catalyst for Hg^0^ oxidation, while HCl plays an important role in the oxidation process [20]. However, the content of Cl in Chinese coal (63–318 mg/kg) is very low, and thus, the even lower content of HCl in flue gas leads to catalysts’ lack of Hg^0^ oxidation activity [21]. Accordingly, it is necessary to study the oxidation of Hg^0^ in the absence of HCl and improve the oxidation ability of Hg^0^ when the catalyst is installed in flue gas conditions with insufficient HCl content.

As is shown in recent work, CeO_2_ can catalyze the oxidation of Hg^0^ in flue gas condition without HCl and obtain a high efficiency of Hg^0^ oxidation with the aid of oxygen [7,22,23]. Li et al. synthesized a series of CeO_2_–TiO_2_ catalysts by an ultrasound-assisted impregnation method. They found that the introduction of 4% O_2_ into the gas flow containing SO_2_ and NO resulted in an Hg^0^ oxidation efficiency as high as 99.9% [7]. He and his co-workers studied the reactivity of CeO_2_ catalyst based on pillared clay(PILC)–TiO_2_, and the results demonstrated that 15% Ce/Ti-PILC catalyst had the best Hg^0^ oxidation efficiency (88.6%) at 300 °C under a 5% O_2_ + N_2_ atmosphere [22]. Zhao et al. studied the catalytic effect of a CeO_2_-doped V_2_O_5_–WO_3_/TiO_2_ catalyst under flue gas conditions without HCl and found that the highest oxidation efficiency of Hg^0^ was 88.93% at the optimum temperature of 250 °C. XPS results showed that Hg^0^ and HgO are the main mercury species on the catalyst surface, indicating that Hg^0^ was oxidized to HgO by the oxygen atoms on the surface of the catalyst, and the oxidation process should follow the Mars–Maessen mechanism [23].

It is generally believed that there are four main mechanisms of Hg^0^ heterogeneous oxidation hitherto, i.e., the Deacon process, the Mars–Maessen mechanism, the Langmuir–Hinshewood mechanism, and the Eley–Rideal mechanism [24]. Recent DFT research indicates that the heterogeneous oxidation of Hg^0^ and HCl on the catalyst surface follows the Langmuir–Hinshewood mechanism [10,25,26,27,28]. Nevertheless, to the best of our knowledge, few theoretical calculations were reported to investigate the reaction mechanism between Hg^0^ and oxygen on the catalyst surface in detail, and there is no quantum chemistry study concerning the Hg + O_2_ → HgO progress on the CeO_2_ (111) surface to date. Based on previous work, density functional theory calculations are performed in this study to investigate the mechanism of mercury adsorption and oxidation by oxygen on the CeO_2_ (111) surface, and the reaction pathway, energy barriers, and transition state configurations of interaction between mercury and oxygen are also studied. The objective of this work is to provide theoretical guidance for the Hg^0^ catalytic oxidation without HCl.

## 2. Computational Details

### 2.1. Catalyst Models

**Figure 1:**
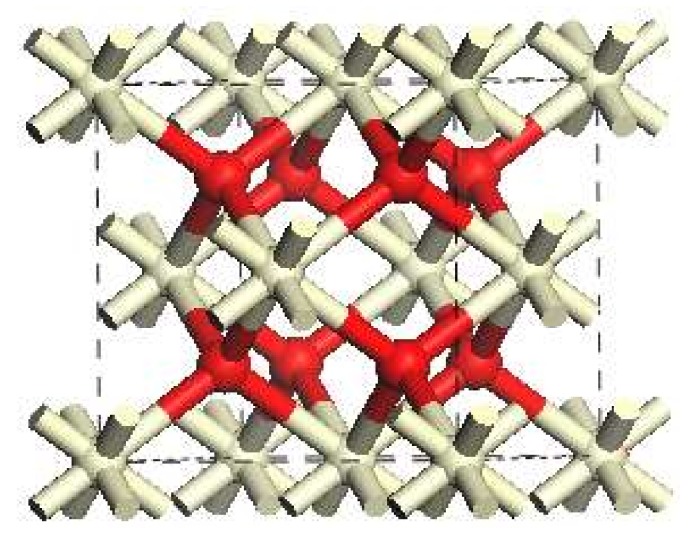
CeO_2_ unit cell: red ball = O atom; white ball = Ce atom.

The crystal structure of CeO_2_ is of a cubic fluorite structure with a space group Fm-3m. It contains four Ce atoms and eight O atoms in a primary cell, as shown in Figure 1.

**Figure 2:**
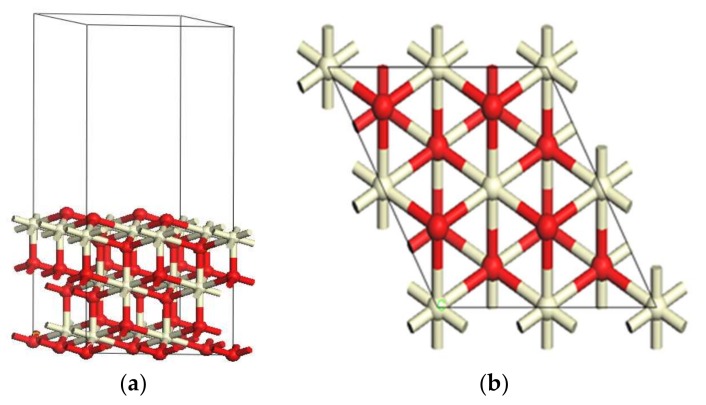
CeO_2_ (111) surface model: (**a**) Side view; (**b**) top view.

Previous research indicates that CeO_2_ (111) is a typical low index surface with the most stable thermodynamic properties, and also has the closest physical and chemical properties to a real crystal surface [20]. Hence the CeO_2_ (111) surface was constructed by cleaving the optimized unit cell. In this work, p (2 × 2) and p (3 × 3) supercell periodic slab models with nine atomic layers were constructed. The bottom six layers were fixed in their bulk positions and the top three layers were fully relaxed for geometry optimization. A 12 Å-thick vacuum region was set so that the energy effect of interactions between slabs can be neglected. The energy effect of neighboring Hg atoms was tested by comparing the adsorption energies of Hg on p (2 × 2) and p (3 × 3) surfaces. The results indicate that the geometry parameters and adsorption energies of Hg adsorption are close (−5.71 kJ/mol vs. −6.76 kJ/mol), which is consistent with previous work [20]. Therefore, the p (2 × 2) model is used to simulate the CeO_2_ surface. The constructed CeO_2_ (111) surface model is depicted in Figure 2.

### 2.2. Computational Method

All density functional theory calculations in this study were performed using DMol^3^ code [29]. The exchange-correlation potential was calculated by the Perduw-Burke-Ernzerhof (PBE) function in a generalized gradient approximation (GGA) scheme [30,31]. The core electrons of Hg and Ce atoms were treated by the DFT semi-core pseudopotential (DSPP) method, in which the relativistic effect of core electrons was concerned, while the core electrons of O atoms were treated by the all-electron method [32]. A 4.5 Å global orbital cutoff was selected with a smearing value of 0.005 Ha to accelerate the convergence of calculations. The molecular orbitals are expanded by a double numerical basis set with polarization functions (DNP).

To properly account for the band structure of ceria, Hubbard U corrections to the f electrons have been applied in some calculations on the CeO_2_ surface [33,34]. However, previous results indicate that plain DFT calculations can provided a reasonable prediction of reduction energies, even better than that from DFT + U [34,35,36,37,38]. Moreover, the effect of U correction on reactions on stoichiometric CeO_2_ (111) surface is relatively not significant [39,40], which makes this approach less cost-effective. Hence, the DFT+U method was not considered in this study.

3 × 3 × 1 Monkhorst–Pack k-point sampling was selected for Brillouin zone integration during geometric optimization calculation. The convergence criteria of energy, force, and displacement are less than 10^−5^ Hartree, 0.002 Ha/atom and 0.005 Å, respectively.

To confirm the accuracy of the above parameters, a 4 × 4 × 4 k point mesh was first performed for geometry optimization of CeO_2_ unit cell. The optimized bulk lattice parameters (a = b = c = 5.485 Å) are in good agreement with the experimental values and calculated values reported in the literature [41]. The deviation is found to be minimal, which suggests that the calculations are reliable. The adsorption energy on CeO_2_ (111) surface (*E_ads_*) is defined as follows:

(1) $$E_{ads}=E_{adsorbate-substrate}-E_{adsorbate}-E_{substrate},$$

where $E_{adsorbate-substrate}$ represents the adsorption configuration on CeO_2_ (111) surface, $E_{adsorbate}$ and $E_{substrate}$ represent the total energy of adsorbate and CeO_2_ (111) surface, respectively. As shown in Equation (1), a negative value of adsorption energy refers to an endothermic reaction, while a positive value refers to an exothermic reaction. In addition, the more negative the adsorption energy, the more stable the adsorption configuration is.

The oxidation pathway of Hg^0^ includes intermediate (IM), transition state (TS) and final state (FS). All TSs were obtained by the complete linear synchronous transit and quadratic synchronous transit (LST/QST) method [42]. Vibrational frequencies were calculated at the optimized geometries to identify the nature of the stationary points (no imaginary frequency) and the transition state (only one imaginary frequency). The energy barriers of each reaction pathways are defined by the following equation:

(2) $$E_{barrier}=E_{transition\;state}-E_{intermediate},$$

where $E_{transition\;state}$ and $E_{intermediate}$ denote the total energies of transition state and intermediate, respectively.

## 3. Results and Discussion

### 3.1. Adsorption of Hg^0^ on the CeO_2_ (111) Surface

**Figure 3:**
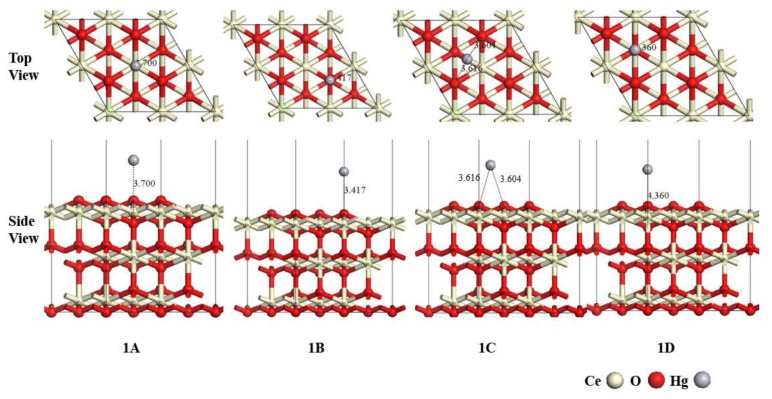
Adsorption configurations of Hg^0^ on the CeO_2_ (111) surface.

Hg^0^ adsorption on the catalyst surface is the first step in heterogeneous mercury oxidation. All possible adsorption sites were considered on the CeO_2_ (111) surface and four stable configurations (Ce site, O-top site, O-sub site, and O-bridge site) are shown in Figure 3 as 1A, 1B, 1C, and 1D. The adsorption energy, Mulliken charge of Hg atom and corresponding geometry parameters of 1A, 1B, 1C, and 1D are listed in Table 1.

According to Figure 3 and Table 1, all the adsorption energy values are negative, indicating that the Hg^0^ adsorption on the CeO_2_ (111) surface is an exothermic process. In configurations 1B, 1C, and 1D, the adsorption of Hg atoms form Hg–O bonds with surface O atoms with lengths of 3.053 Å, 3.616/3.604 Å, and 4.360 Å, respectively. The adsorption energies are −1.411 kJ/mol, −5.30 kJ/mol, and −5.25 kJ/mol, respectively, suggesting a weak interaction. About 0.008 and 0.011 e charges of Hg atom are transferred to the surface in adsorption, suggesting that few electrons are transferred to the substrate, which is consistent with the results of adsorption energy. Regarding configuration 1A, the bond length between Hg^0^ and surface Ce atom is 3.797 Å, and the Mulliken charge of the Hg atom is about 0.012 e. Meanwhile, it has the most negative adsorption energy of −5.71 kJ/mol. Therefore, it is the most stable configuration for Hg^0^ adsorption and the adsorption mechanism is physisorption. The calculated results agree well with previous theoretical research [20].

**Figure 4:**
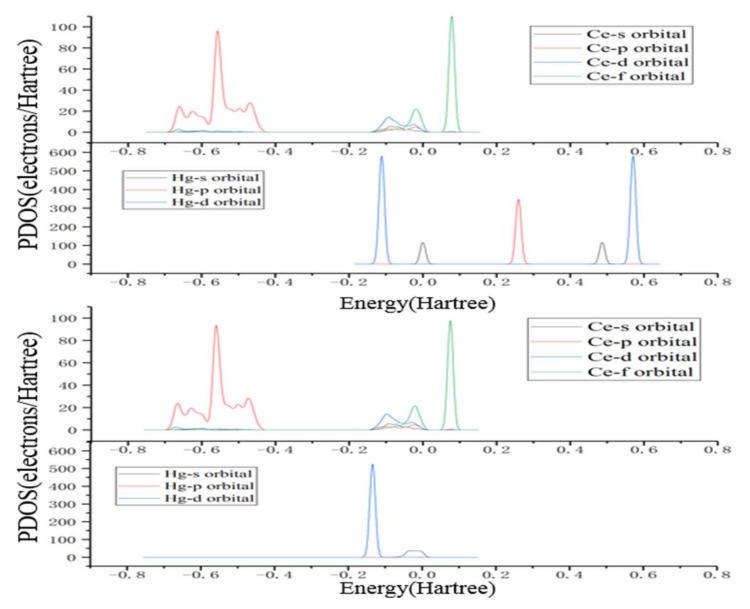
PDOS of Hg^0^ adsorption on the CeO_2_ (111) surface before and after adsorption.

To further investigate the interaction between the Hg atom and the CeO_2_ (111) surface during adsorption, the partial density of states (PDOS) analysis of Hg and Ce atoms are performed in the most stable adsorption configuration of 1A. The PDOS results of Hg pre-adsorption, Hg post-adsorption, Ce pre-adsorption, and Ce post-adsorption are presented in Figure 4. As is depicted in Figure 4, for pre-adsorption, the Hg s-orbital is occupied at 0.49 Ha and the Fermi level. The unoccupied p-orbital shows a single peak at 0.22 Ha, while the Hg d-orbital is occupied at −0.13 and 0.57 Ha. After Hg adsorption, all orbitals of the Hg atom are shifted to the lower energy level with s- and p-orbitals broadened and there is a decrease in energy due to the charge transfer from the Hg atom to the surface Ce atom. Meanwhile, the disappearance of the Hg d-orbital located at 0.57 Ha indicates a strong interaction between the Hg atom and the CeO_2_ (111) surface. As for the energy bands located between −0.7 Ha and 0.1 Ha of the surface Ce atom, an unapparent change occurs after adsorption in all orbitals. It can be concluded from the above analysis that Hg has a strong interaction with the CeO_2_ (111) surface after adsorption. Hg orbitals change significantly, while no apparent change occurs for the surface Ce atom, indicating that the CeO_2_ (111) surface can remain stable after Hg^0^ adsorption.

### 3.2. Adsorption of HgO on the CeO_2_ (111) Surface

**Figure 5:**
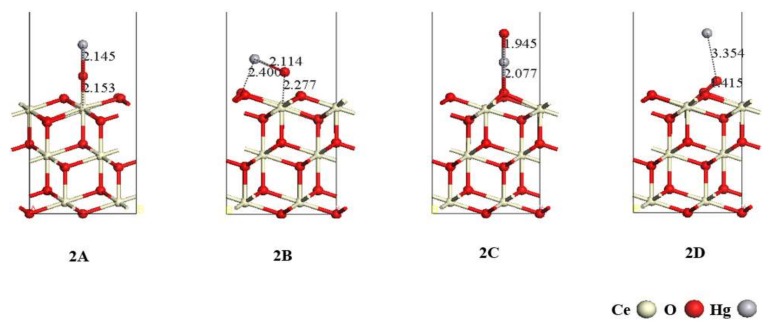
HgO adsorption configurations on the CeO_2_ (111) surface.

Heterogeneous oxidation of Hg^0^ will form HgO on the catalyst surface. Similar to Hg^0^, the adsorption behavior of HgO was also investigated on the CeO_2_ (111) surface. All possible adsorption orientations, including parallel and perpendicular, were considered. After geometry optimization, four stable configurations, 2A, 2B, 2C, and 2D, are presented in Figure 5 and the corresponding adsorption energy and geometric parameters are given in Table 2. The configuration 2D with an adsorption energy of −198.37 kJ/mol is the most stable. HgO molecule is dissociatively adsorbed on the CeO_2_ (111) surface with a breakage of the Hg–O bond. The O atom of the HgO molecule forms a 1.415 Å O–O bond with the O-top atom on the CeO_2_ (111) surface. In the configuration 2A, the O-end of HgO is vertically adsorbed on the Ce atom of the CeO_2_ (111) surface, forming a 2.153 Å Ce–O bond. The length of Hg–O slightly decreases from 2.208 Å to 2.145 Å, and the adsorption energy of 2A is 69.90 kJ/mol. In terms of the configuration 2B, HgO molecules are adsorbed on the CeO_2_ (111) surface in an approximately parallel manner. The length of the Hg–O bond is 2.277 Å, while the distances between Hg, O, and the CeO_2_ (111) surface are 2.114 Å and 2.400 Å, respectively. The corresponding adsorption energy is −101.99 kJ/mol. As for the 2C configuration, the Hg end of HgO molecule is adsorbed perpendicularly to the O-top atom on the surface with an adsorption energy of −163.81 kJ/mol. The length of Hg–O decreases to 1.945 Å and the Hg–O-top distance is 2.077 Å.

Investigation of HgO adsorption on the CeO_2_ (111) surface indicates that the adsorption manner of HgO is chemisorption due to its strong adsorption energy. Hence, it is difficult for HgO to separate from the CeO_2_ (111) surface. In the most stable adsorption configuration, 2D, HgO dissociates on the surface with an adsorption energy of −198.37 kJ/mol; the Hg–O bond in the HgO molecule breaks and the O atom is bonded with the surface oxygen. Apart from 2D, 2C has the most negative adsorption energy of −163.81 kJ/mol. Based on the calculated results, the interaction of HgO with oxygen on the CeO_2_ (111) surface is relatively strong, which is instructive for the consideration of reaction sites on the surface.

### 3.3. Hg^0^ Oxidation Mechanism on the CeO_2_ (111) Surface

The surface oxygen on the catalyst plays a crucial role in the Hg^0^ heterogeneous oxidation process to form HgO. Its consumption can be replenished by gas phase O_2_ and Hg^0^ oxidation by surface oxygen is regarded as a Mars–Maessen process [43]. The Mars–Maessen mechanism can be illustrated by Equations (3)–(7) [24].

(3) A(g)→A(ads),

(4) $$A\left(ads\right)+M_{x}O_{y}\to AO\left(ads\right)+M_{x}O_{y-1},$$

(5) MxOy−1+12O2→MxOy,

(6) AO(ads)→AO(g),

(7) $$AO\left(ads\right)+M_{x}O_{y}\to AM_{x}O_{y+1}.$$

**Figure 6:**
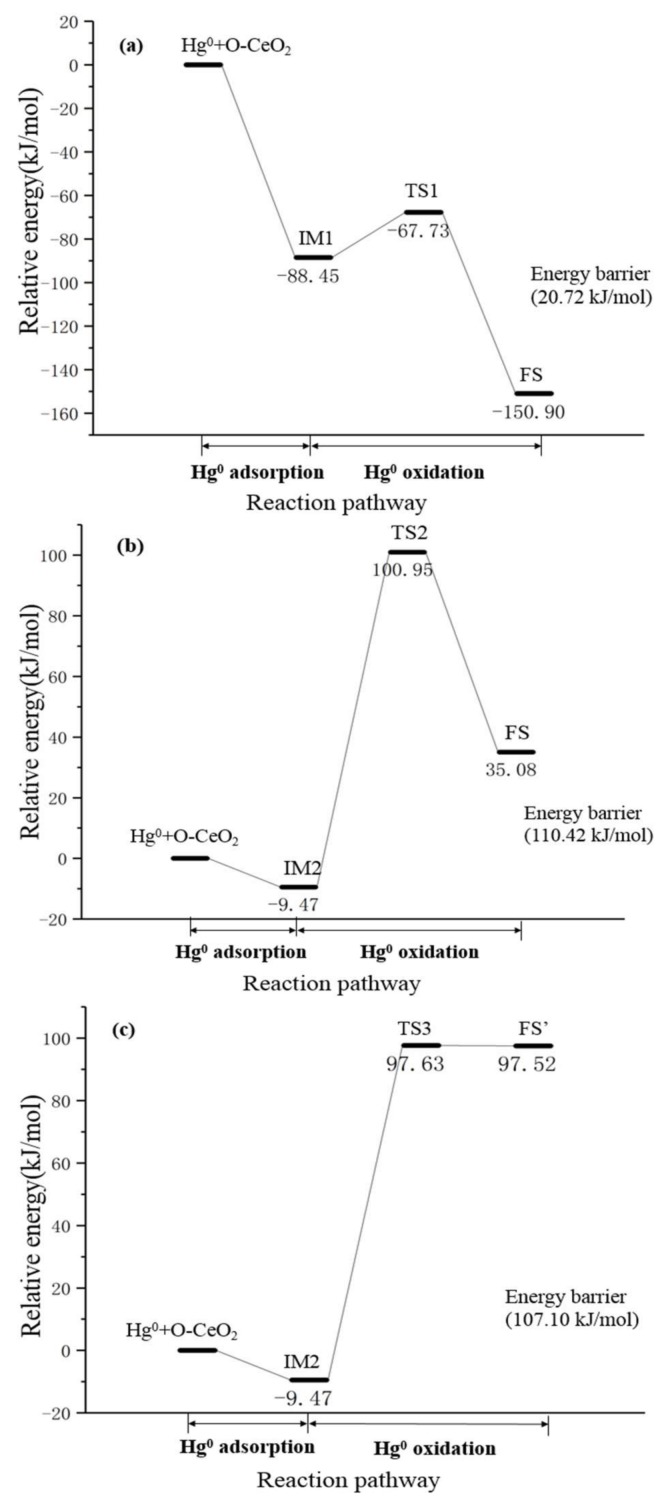
Reaction pathways and energy profiles of oxidation between Hg^0^ and O_2_ over the CeO_2_ (111) surface: (**a**) pathway I; (**b**) pathway II; (**c**) pathway III.

**Figure 7:**
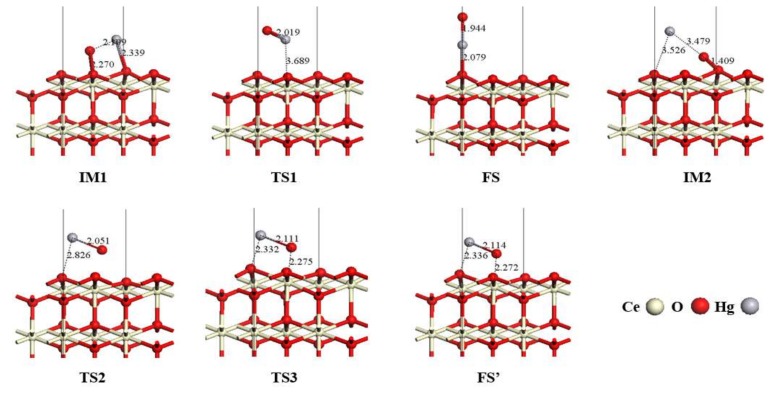
Configurations of intermediates, transition states and final states in Hg^0^ oxidation pathways on the CeO_2_ (111) surface.

The Hg atom is initially adsorbed on the surface and then oxidized by the surface oxygen to form HgO. Based on the above calculations, the configurations of intermediate and final state can be determined according to the adsorption behaviors of Hg species. Furthermore, three possible reaction pathways are obtained by transition state search and frequency calculation verification. The proposed reaction pathways are given in Figure 6 with relative energy barriers. The optimized geometric configurations of intermediate (IM), transition state (TS) and final state (FS) in each reaction pathway are depicted in Figure 7.

In the case of pathway I, shown in Figure 6a, during the first stage, Hg^0^ and surface O atoms are firstl adsorbed on O-top site of the CeO_2_ (111) surface, forming IM1. The adsorption of Hg atom is a physisorption process and a barrierless exothermic reaction with exothermicity of −88.45 kJ/mol. In IM1, oxygen is adsorbed on the surface O-top atom with a 2.270 Å O–O_top_ bond, while the Hg atom is adsorbed in a physical manner, as mentioned above, and the length of the Hg–O_top_ bond is 2.339 Å. The distance between Hg^0^ and surface oxygen is 2.109 Å, which is larger than the Hg–O bond length of the gas-phase HgO molecule [43]. Then Hg^0^ moves toward the surface O atom and is oxidized by overcoming an energy barrier of 20.7 kJ/mol through transition state TS1. During this process, the distance between the surface O atom and Hg^0^ decreases gradually, i.e., 2.109 Å (IM1) → 2.019 Å (TS1) → 1.944 Å (FS). In TS1, Hg and O atoms move closer to each other, leading to the formation of an Hg–O bond with a length of 2.019 Å. The O atom migrates upward from the surface together with the Hg atom, and the distance of the Hg atom from the surface is 3.689 Å. In FS, Hg^0^ is oxidized to HgO with its Hg atom close to the CeO_2_ (111) surface, and is adsorbed in a vertical manner. The distance between Hg atoms and the surface is 2.079 Å, and the bond length of Hg–O further shortens to 1.944 Å. In this pathway, the reaction of Hg^0^ oxidation is an exothermic process and the reaction heat is −62.5 kJ/mol.

Figure 6b presents the energy profile of pathway II. Similar to pathway I, Hg^0^ is first adsorbed on the CeO_2_ (111) surface through a barrierless physical adsorption process. In this step, the reaction heat was −9.99 kJ/mol. IM2 formed after Hg^0^ was dissociatively adsorbed on CeO_2_ (111) surface. In IM2 configuration, adsorbed oxygen on the Otop site of CeO_2_ (111) surface forms a 1.409 Å O–O bond. The distance between Hg and surface O atom is 3.479 Å, which is much larger than the bond length of the gas-phase HgO molecule. Consequently, an oxidation reaction between the adsorbed Hg atom and the surface oxygen occurs in the IM2 configuration, during which the Hg atom approaches surface oxygen and HgO molecule forms on the surface O-site via IM2 → TS2 → FS. In TS2, the surface O atom is stripped by the Hg atom from the CeO_2_ (111) surface, resulting in the breakage of the surface O–O bond. The Hg atom also moves closer to the surface, from 3.526 Å to 2.826 Å. The distance between the surface O atom and Hg^0^ is similarly shortened gradually, i.e., 3.479 Å (IM2) → 2.051 Å (TS2) → 1.944 Å (FS), indicating the formation of an Hg–O bond. Subsequently, the HgO molecule in the TS2 configuration turns over and the horizontal adsorption configuration with the HgO molecule’s O atom near the surface transforms into a vertical adsorption configuration with the Hg atom near the CeO_2_ (111) surface. The whole reaction pathway is endothermic by 44.6 kJ/mol, with an energy barrier of 116.4 kJ/mol.

In addition, after the exothermic physisorption step with an exothermicity of −9.99 kJ/mol on the CeO_2_ (111) surface, IM2 configuration can be oxidized to FS’ via another potential pathway, which is noted as pathway III. The energy profile of reaction pathway III is presented in Figure 6c. In TS3, the surface oxygen and adsorbed Hg move toward each other, shortening the distance from 3.479 Å to 2.111 Å, which leads to the formation of the Hg–O bond. The distances between the Hg and O atoms and the CeO_2_ (111) surface are 2.332 Å and 2.275 Å, respectively. Finally, the FS’ is formed, in which HgO molecular is adsorbed in a parallel fashion on the surface. The length of the Hg–O bond is stabilized at 2.114 Å, while the distances between the Hg and O atoms and CeO_2_ (111) surface exhibit a tiny change and are 2.336 Å and 2.272 Å, respectively. This process is endothermic by 107.0 kJ/mol, with an energy barrier of 107.1 kJ/mol. It is noteworthy that since the coordinates of the saddle point on the potential energy surface are very close to the coordinates of the final state, the geometry configuration of TS3 is analogous to that of FS’, which makes the energy barrier of pathway III close to reaction heat.

In the above three pathways, since Hg^0^ adsorption on the surface is a barrierless process, the formation of HgO is the rate-determining step of the entire oxidation reaction. In addition, pathway I not only has the lowest energy barrier but also is an exothermic process. Hence, the Hg^0^ oxidation by oxygen on CeO_2_ (111) surface tends to occur through pathway I. In regard to other mercury heterogeneous oxidation mechanisms except the Mars–Maessen mechanism, Liu et al. studied the oxidation between Hg^0^ and HCl on the CeO_2_ (111) surface, identifying the rate-determining step as the formation of HgCl_2_ (Hg^0^ + HCl → HgCl_2_); this step followed the Langmuir–Hinshelwood mechanism [20]. Comparing the reaction pathways proposed in this work with the study by Liu et al. on the reaction between Hg^0^ and HCl, it is found that the energy barrier of pathway I, 20.7 kJ/mol, is lower than that of HgCl_2_ formation (56.92–78.05 kJ/mol). This suggests that the Mars–Maessen oxidation of Hg^0^ by oxygen on the CeO_2_ (111) surface can be of the same reactivity as the Langmuir–Hinshelwood oxidation of Hg^0^ by HCl.

Furthermore, the energy barrier of HgO forming on the CeO_2_ (111) surface is also compared with that on other materials, such as V_2_O_5_/TiO_2_ (001) and MnFe_2_O_4_ (100) [43,44]. In both systems, the oxidation of Hg^0^ by oxygen follows the Mars–Maessen mechanism. The barrierless adsorption of Hg^0^ is the first step and the formation of the HgO molecule is the rate-determining step, which is consistent with the results calculated in this study. It is found that CeO_2_ is superior at catalyzing the Hg^0^ oxidation by oxygen due to its lower energy barrier than the V_2_O_5_/TiO_2_ (001) system (143.9–151.2 kJ/mol) and the MnFe_2_O_4_ (100) surface (76.07–200.37 kJ/mol). Therefore, it can be concluded that, whether as a catalyst for Hg^0^ oxidation or as an adjuvant to modify the V–Ti-based catalyst, CeO_2_ exhibits excellent Hg^0^ oxidation performance, especially with insufficient HCl content in flue gas when burning low-rank coal. Based on the above results, it can be speculated that CeO_2_ can efficiently catalyze the oxidation of Hg^0^ by oxygen in flue gas and can be a promising catalytic material for flue gas mercury removal.

## 4. Conclusions

Mercury adsorption and oxidation mechanisms with oxygen on the CeO_2_ (111) surface were investigated using DFT calculations for the first time in this study. The adsorption manners of Hg species were studied first. Hg^0^ adsorbs on the CeO_2_ (111) surface in a physical adsorption manner with a −1~−5 kJ/mol adsorption energy, while HgO molecular is chemisorbed on the surface with its adsorption energy in the range of −69.90~−198.37 kJ/mol. Density of state analysis for Hg and Ce atoms indicates that Hg^0^ significantly interacts with the surface Ce atom and the adsorption configuration is stable. DFT calculations indicate that the reaction between Hg^0^ and surface oxygen on CeO_2_ (111) follows the Mars–Maessen mechanism. In the first step Hg^0^ is adsorbed on the surface through an exothermic process with no energy barriers; during the second stage, the adsorbed Hg atom is oxidized by the surface oxygen, which is subsequently replenished by the gas phase O_2_. Three possible reaction pathways are proposed for the abovementioned oxidation mechanism; among them the pathway I is exothermic and has the lowest energy barrier, while the others have relatively high energy barriers and are endothermic, which suggests that pathway I is the most favorable oxidation route. The rate-determining steps are the formation of the HgO molecule in each pathway.

To better evaluate the oxidation ability of the CeO_2_ + O_2_ system, energy barriers are compared with relative systems including CeO_2_ + HCl, V_2_O_5_ + O_2_, and MnFe_2_O_4_ + O_2_. The comparative analysis shows that the energy barrier of the oxidation reaction between Hg^0^ and oxygen on CeO_2_ (111) surface is lower than that of the reaction of Hg^0^ with HCl, and is also lower than those of other materials, which helps to prove that CeO_2_ is an active catalytic material with high Hg^0^ oxidation ability. In conclusion, the CeO_2_-based catalyst, which can enhance the oxidation rate of Hg^0^ in flue gas, may have promising application prospects for mercury removal, especially in low-rank coal-fired flue gas with a low HCl concentration.

## Figures and Tables

**Table 1:** The adsorption energy, geometry parameters and Mulliken charge for Hg^0^ adsorption on CeO_2_ (111) surface.

Configurations	*E_ads_* (kJ/mol)	R_X-Hg_ (Å) *	Q_Hg_ (e)
1A	−5.71	3.700	0.012
1B	−1.54	3.417	0.008
1C	−5.30	3.616/3.604	0.009
1D	−5.25	4.360	0.011

* X denotes surface atom on the CeO_2_ (111) surface.

**Table 2:** The adsorption energy, geometry parameters, and Mulliken charge for Hg^0^ adsorption on the CeO_2_ (111) surface.

Configurations	*E_ads_* (kJ/mol)	R_X-Hg_ (Å) *	R_O-Hg_ (Å)	R_X-O_ (Å)
2A	−69.90	-	2.145	2.153
2B	−101.99	2.114	2.277	2.400
2C	−163.81	2.077	1.945	-
2D	−198.37	-	3.354	1.415

* X denotes surface atom on the CeO_2_ (111) surface.
